# Alcohol Consumption, Blood Lead Levels and Aggressive Behaviour Among Adults Living Near Gold Mine Tailings in Gauteng, South Africa

**DOI:** 10.1007/s12403-026-00807-7

**Published:** 2026-09-07

**Authors:** Tahira Kootbodien, Renee Street, Yonela Mkunyana, Tanya Haman, Nisha Naicker, Angela Mathee

**Affiliations:** 1 Environment and Health Research Unit, South African Medical Research Council, Francie van Zyl Road, Tygerberg, Cape Town, South Africa; 2 Centre for Environmental and Occupational Health Research, School of Public Health and Family Medicine, University of Cape Town, Observatory, Cape Town, 7925 Western Cape South Africa; 3 Division of the National Health Laboratory Service, National Institute of Occupational Health, 25 Hospital St, Constitution Hill, Johannesburg, 2000 South Africa; 4 Department of Environmental Health, Faculty of Health Sciences, University of Johannesburg, Johannesburg, South Africa; 5 School of Public Health, Faculty of Health Sciences, University of Witwatersrand, Johannesburg, South Africa

**Keywords:** Blood lead levels, Aggression, Mine tailings, Alcohol use, Heavy episodic drinking

## Abstract

Lead (Pb) is a neurotoxin linked to aggression, while alcohol consumption, a major public health concern in South Africa, may also increase aggressive behaviour. This study examined associations between blood Pb levels and aggression in adults, and whether alcohol modified this relationship. A cross-sectional study was conducted among 199 adults, living at varying distances from gold mine tailings in Gauteng. Blood Pb were log-transformed and examined in relation to environmental exposures (proximity to roads and mine tailings), behavioural factors (smoking, soil ingestion), and soil Pb levels. Alcohol consumption was categorised as none, 1–4 drinks and ≥ 5 drinks per occasion (heavy episodic drinking). Aggression was measured using the 29-item Buss Perry Aggression Questionnaire (BPAQ), including anger, hostility, verbal, and physical aggression. Linear regression models were estimated for each aggression outcome, adjusting for age, alcohol use, blood Pb, and interactions between blood Pb and alcohol. Mean blood Pb was 2.4 ± 1.8 µg/dL (range 0.5–12.2 µg/dL), with 9.7% exceeding 5 µg/dL and 18.7% exceeding 3.5 µg/dL. Higher blood Pb was associated with soil ingestion (β = 0.29, *p* = 0.017), higher soil Pb (β = 0.08, *p* = 0.001), tobacco use (β = 0.20, *p* = 0.031) and household dust (β = 0.13, *p* = 0.043). Heavy episodic drinking was associated with higher total BPAQ scores (β = 19.7, *p* < 0.001) and all subscales. Blood Pb showed marginal associations with verbal and physical aggression, and alcohol did not modify the Pb-aggression relationship. In this population, heavy episodic drinking, rather than Pb exposure, was the main factor linked to increased aggression. Addressing harmful alcohol use may provide an opportunity to reduce a modifiable risk factor for aggression, while environmental Pb surveillance and remediation remain important in communities with ongoing Pb exposure.

## Highlights

Heavy episodic drinking was the most consistent predictor of aggression across all subscales.

Pb exposure was marginally associated with aggression, with small effects across subscales.

Reducing heavy episodic drinking may be an effective strategy to prevent aggression, even in populations exposed to environmental Pb.

## Introduction

Lead (Pb) exposure and harmful alcohol use are both recognised determinants of aggressive behaviour (Fritz et al. 2023; Reyes Sánchez et al. 2022; Shaffer et al. 2022; Sontate et al. 2021). In children, exposure to Pb, a neurotoxin, has been associated with reduction in intelligence quotient (IQ) scores (Jakubowski 2011; Lanphear et al. 2019) and behaviour changes such as shortened concentration spans, poor school performance (Bellinger 2008), delinquency (Needleman et al. 1996, 2002) and aggressive and antisocial behaviour (Shaffer et al. 2022; Wright et al. 2008). Among adults, childhood Pb exposure has been associated with violent and criminal behaviour later in life (Beckwith et al. 2021; Wright et al. 2021). In South Africa, studies have found associations between elevated blood Pb levels and increased aggression, including higher scores for anger, hostility, and rule-breaking behaviour among adolescents, suggesting that lead neurotoxicity may contribute to aggressive behaviour in high Pb exposure communities (Naicker et al. 2012; Nkomo et al. 2018).

Alcohol use is a major contributor to the global burden of disease, accounting for an estimated 2.6 million deaths worldwide in 2019, with the highest levels of alcohol-attributable deaths per 100 000 persons observed in the WHO African Region (World Health Organization, 2024). South Africa has among the highest rates of alcohol consumption globally, which is implicated in nearly half of all non-natural deaths and imposes a substantial economic burden (Matzopoulos et al. 2014, 2022). Findings from brain studies have reported that long term alcohol use can result in decreased cortical thickness in brain regions involved in self-control, decision-making, and emotional processing (Daviet et al. 2022). While the underlying mechanisms are not fully understood (Chermack and Giancola 1997), these structural changes may help explain the increase in aggressive behaviour after alcohol use.

Legacy gold mine tailings in the Witwatersrand contain a mixture of potentially toxic metals, including lead (Pb), arsenic, cadmium, mercury and uranium (Chetty et al. 2021). Windblown dust from these tailings has been associated with elevated soil and blood lead concentrations in nearby communities, supporting lead as an important environmental exposure in this setting (Mathee et al. 2022). Pb exposure has long been associated with neurotoxic effects that impair impulse control and heighten aggression (Beckwith et al. 2021; Bellinger 2008), while alcohol consumption can amplify aggressive tendencies (Sontate et al. 2021). In communities where exposure to environmental Pb is elevated and alcohol misuse is widespread, these risk factors may act synergistically to heighten levels of aggression, adding to South Africa’s high burden of violence-related morbidity and mortality. In this study, we hypothesised that higher blood Pb levels are associated with increased aggression among adults living near gold mines and mine tailings in Gauteng, South Africa, and this relationship is further exacerbated by alcohol consumption.

## Methods

### Study Population

**Fig. 1:**
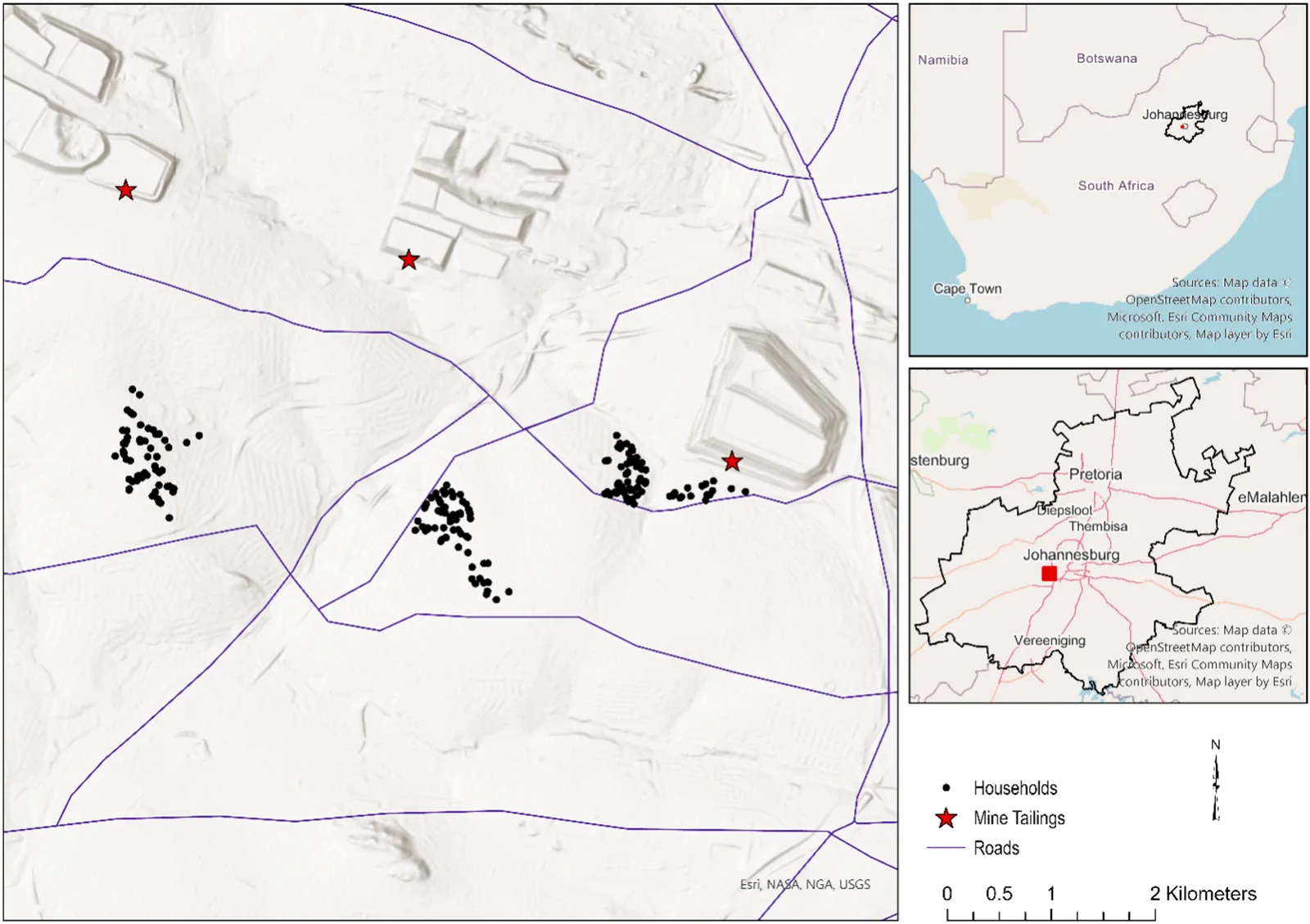
Map of study population showing the proximity of households to gold mine tailings and major roads in Gauteng province, South Africa

We conducted a cross-sectional household survey of adults aged 18 years and older residing in five communities located in Gauteng Province, South Africa (Fig. 1). These communities (Diepkloof, Noordgesig, Orlando East, Orlando West and Meadowlands East) are situated near gold mine tailings and are known to have elevated environmental levels of heavy metals, including Pb (Coetzee et al. 2006). Households were selected using convenient sampling, and households were eligible if they had lived in the area for at least three years. This study follows from a previously published household survey conducted in the same geographical area, which assessed metal exposure from mine tailings and their impact on health and behavioral outcomes in children (Mathee et al. 2022; Zupunski et al. 2023).

### Study Procedure and Measures

A research team consisting of trained fieldworkers and research nurses conducted the interviewer administered questionnaire and collected information on sociodemographic characteristics (e.g., sex, age, education levels, employment status and housing conditions), health-related behaviour questions (smoking habits and alcohol use) and self-reported health outcomes (past medical history). Household socioeconomic status was assessed using 12 binary household asset indicators (e.g., radio, television, refrigerator, computer, telephones, internet access, and child’s room/study).

Venous blood (approximately 5 ml) samples were collected from all consenting study participants by registered nurses. Blood samples were collected into sterile test tubes containing ethylenediaminetetraacetic acid (EDTA) and kept under refrigeration prior to being transferred to an accredited laboratory for Pb analysis. Blood Pb samples were analysed using ICP-MS (Inductively Coupled Plasma - Mass Spectrometry) using an Agilent 7900 ICP-MS instrument (Agilent Technologies, Santa Clara, California, USA), in the targeted analysis mode. Quality assurance was performed by analysing three levels of certified reference materials (Trace Elements in Whole Blood L-1, L-2 and L-3, Sero AS, Norway) after every 10 samples. The laboratory participated in the Royal College of Pathologists of Australasia External Quality Assurance (RCPA EQA) program, with proficiency testing conducted 12 times per year.

*Outcome measures*: Aggressive behaviour was assessed using the Buss-Perry Aggression Questionnaire (BPAQ), a 29-item self-report instrument that measures aggression in adults (Buss and Perry 1992). The BPAQ evaluates aggression across four related but distinct domains: anger, hostility, verbal, and physical aggression. The participants answered statements on a 5-point Likert scale, ranging from 1 (extremely uncharacteristic of me) to 5 (extremely characteristic of me). Each domain or subscale was summed, with the total aggression score calculated as the sum of all items. Higher scores indicate increased aggressive behaviour. The Cronbach’s α of the BPAQ in the study population was 0.846, suggesting high internal consistency.

### Statistical Analysis

Descriptive statistics were presented for all variables, and where appropriate, means (or geometric means) ± standard deviations (SD) are presented. Categorical variables are reported as frequencies and percentages. The limit of detection (LOD) for blood Pb levels was 0.5 µg/dL. Bivariate analysis included Spearman correlations of Pb concentrations and BPAQ scores, comparisons of mean aggression scores across blood Pb categories (< 5 µg/dL vs. ≥5 µg/dL and quartiles) and across alcohol use categories (no alcohol consumption, 1–4 drinks and ≥ 5 drinks at a time), and tests for linear trends. Heavy episodic drinking or binge drinking was defined as consuming more than 5 drinks on a single occasion in the last month. Household socioeconomic status was assessed by using 12 yes/no variables describing ownership of household assets e.g., radio, refrigerator, computer, etc. Responses were combined using principal component analysis, and the first principal component was retained and standardised to create an overall measure of household assets or wealth. The score was divided into quintiles (lowest [1] to highest [5] asset index).

Household GPS coordinates were imported into ArcGIS to calculate the distances from each household to the nearest main road and the nearest mine tailing. This was done using the Spatial Analyst tool, which identified the closest feature in the reference layer and computed the shortest distance between feature pairs. This was done separately for road and mine tailings layers, with all datasets projected to the same coordinate system (Hartebeesthoek94) to ensure accurate distance estimation (in meters). These GIS-derived proximity measures were used as indicators of potential environmental Pb exposure.

To identify statistically significant spatial clusters of elevated and significantly low soil and blood lead concentrations, Getis-Ord Gi* hotspot analysis was performed. Spatial relationships were modeled using a fixed distance band (Getis and Ord 1992). Statistically significant hotspots were defined as clusters of high Pb concentrations with positive z-scores and p-values corresponding to the 90% (z ≥ 1.65), 95% (z ≥ 1.96), or 99% (z ≥ 2.58) confidence levels. Conversely, statistically significant cold spots were defined as clusters of low Pb concentrations with negative z-scores (z ≤ − 1.65, − 1.96, or − 2.58) at the same confidence levels. Points that did not meet these significance thresholds (*p* > 0.10) were classified as not statistically significant.

Multivariate linear regression models were applied to identify factors associated with log-transformed blood Pb (logPb) exposure, including environmental factors (proximity to mine tailings and to the nearest main road, soil Pb concentrations), behavioural factors (smoking, alcohol use, pica behaviour) and household characteristics. Continuous variables were log-transformed as appropriate, and coefficients estimates were determined using ordinary least squares with 95% confidence intervals. Next, we examined the association between logPb and aggression (total scores and subscales), and tested whether the relationships varied by alcohol use, by including an interaction term (logPb × alcohol), adjusting for age and sex. Vehicle traffic can resuspend Pb previously deposited in roadside soils, allowing legacy Pb contamination to persist for decades after the phase-out of leaded petrol (Resongles et al. 2021). This may represent an additional source of Pb exposure alongside mine tailings in the study area. Distance to main roads was included as a covariate in models examining associations between blood lead level and aggression outcomes (BPAQ total score and anger, hostility, verbal aggression, and physical aggression subscales). Road distance was not significantly associated with BPAQ total or subscale scores (all *p* > 0.20), and its inclusion did not change the magnitude or significance of the blood Pb-aggression associations; it was therefore not included in the final models.

All analyses were conducted in Stata (version 18), and significance was defined at a two-sided *p* < 0.05.

### Ethical Considerations

Written informed consent was obtained from each participant included in the study. Ethical approval was obtained from the Human Research Ethics Committee of the South African Medical Research Council (Protocol number EC001-2/2015).

## Results

### Descriptive Results

The study population characteristics are presented in Table 1. Of the 199 adult participants, the majority were female (87%). The mean age of the participants was 42.9 ± 14.6 years, ranging from 18 to 80 years. The majority of the study population (78%) reported an average monthly household income of less than R5000 (USD 299) and only 15% were employed. Approximately 46% of participants reported smoking, while 48% reported consuming alcohol. Approximately 14% of participants reported heavy episodic or binge drinking i.e. consuming more than five drinks on a single occasion in the last month. The mean blood lead levels were 2.4 ± 1.8 µg/dL, ranging from 0.5 to 12.2 µg/dL, with approximately 10% (19) of the participants’ levels above 5 µg/dL and 18% (36) above 3.5 µg/dL.

**Table 1:** Characteristics of the study population, blood lead levels and reported alcohol use (*n* = 199)

Participant characteristics	Mean ± SD or Frequency (%)
Demographics	
Age	42.4 ± 14.5
Sex	
Female	173 (86.9)
Male	26 (13.1)
Average monthly household income	
R0 - R 1000	40 (21.2)
R1001 - R 5000	109 (58.1)
> R5001	39 (20.7)
Highest level of completed education	
None or primary school	18 (9.6)
High school	154 (81.9)
Tertiary education	16 (8.5)
% Employed	29 (14.6)
Household socioeconomic status (0–5)	2.9 ± 1.4
Age of the home (in years)	54 ± 26
Median distance to nearest mine tailing, in meters (range)	2350.2 (251.3–8344.3)
% Living close to a busy road	118 (57.8)
Median distance to nearest main road, in meters (range)	664.9 (4.9–1551)
Health measures	
Body mass index (BMI)	28.4 ± 8.1
% BMI ≥ 30 (obesity)	71 (35.7)
Hemoglobin (g/dl)	14.1 ± 5.4
Self-reported geophagia practices	28 (14.1)
Exposures	
Current smoker	91 (45.7)
Current alcohol use	95 (47.5)
Frequency of alcohol consumption	
Monthly or less	57/95 (60.0)
2–4 times a month	27/95 (28.4)
2–3 times a week	5/95 (5.3)
≥4 times a week	6/95 (6.3)
Number of alcoholic drinks consumed on a typical day among current alcohol consumers	
1–2 drinks	26/95 (27.4)
3–4 drinks	41/95 (43.2)
5–6 drinks	15/95 (15.8)
7–9 drinks	4/95 (4.2)
> 9 drinks	9/95 (9.4)
% Heavy episodic or binge drinking (≥ 5 drinks per occasion)	27 (13.6)
Blood lead, Pb (µg/dL)	
Mean ± SD	2.4 ± 1.8
Median (range)	2.0 (0.5–12.2)
% ≥ 5 µg/dL	19 (9.5)
% ≥ 3.5 µg/dL	36 (18.1)

### Pb Exposure, Alcohol Consumption and Aggression Scores

**Fig. 2:**
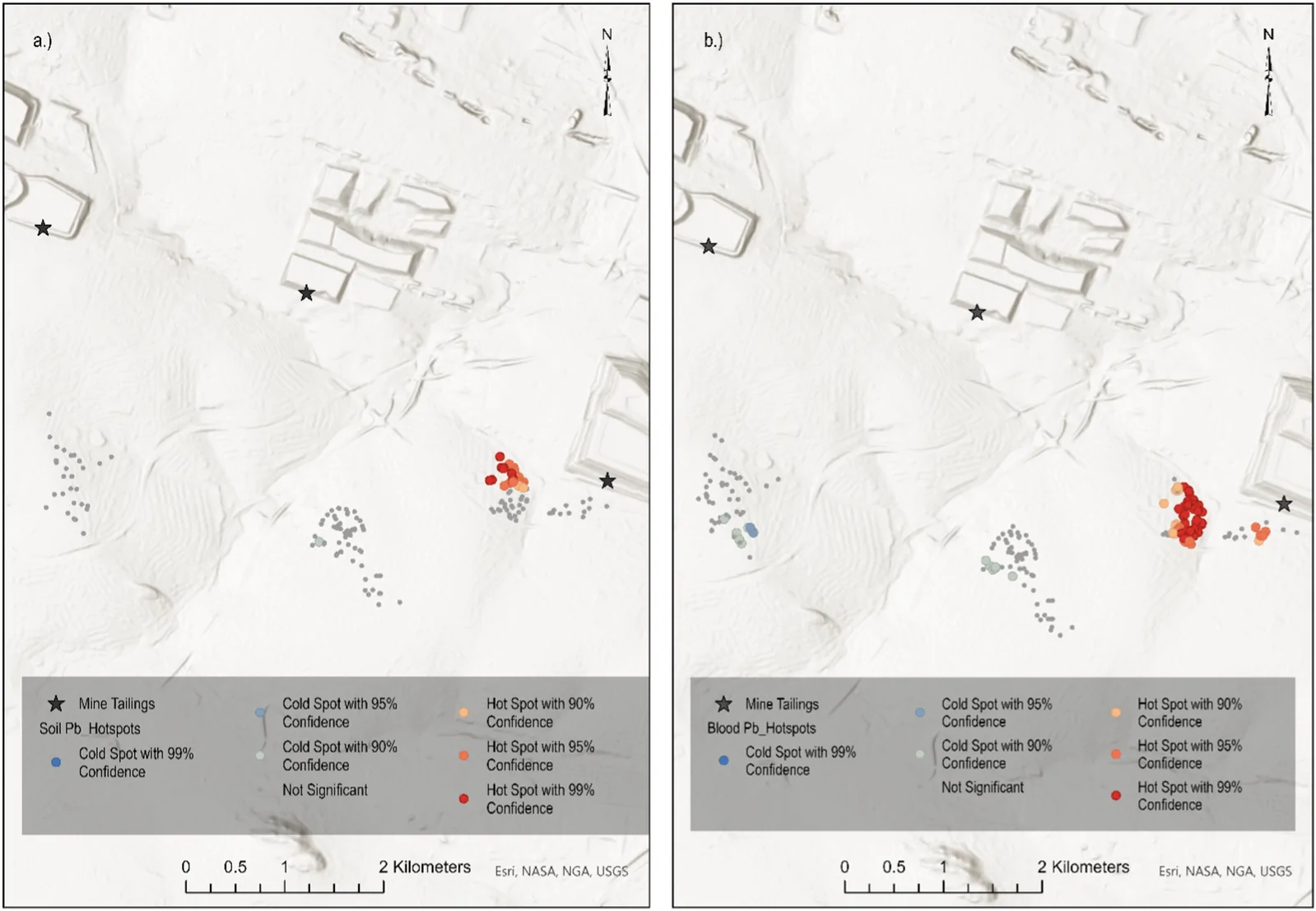
Spatial clustering of soil and blood Pb concentrations around mine tailings using the Getis-Ord Gi statistic. Red circles indicate statistically significant clusters of high soil or blood Pb concentrations (hotspots), while the blue areas indicate clusters of low concentrations (cold spots). Areas shown in grey did not exhibit statistically significant clustering

**Fig. 3:**
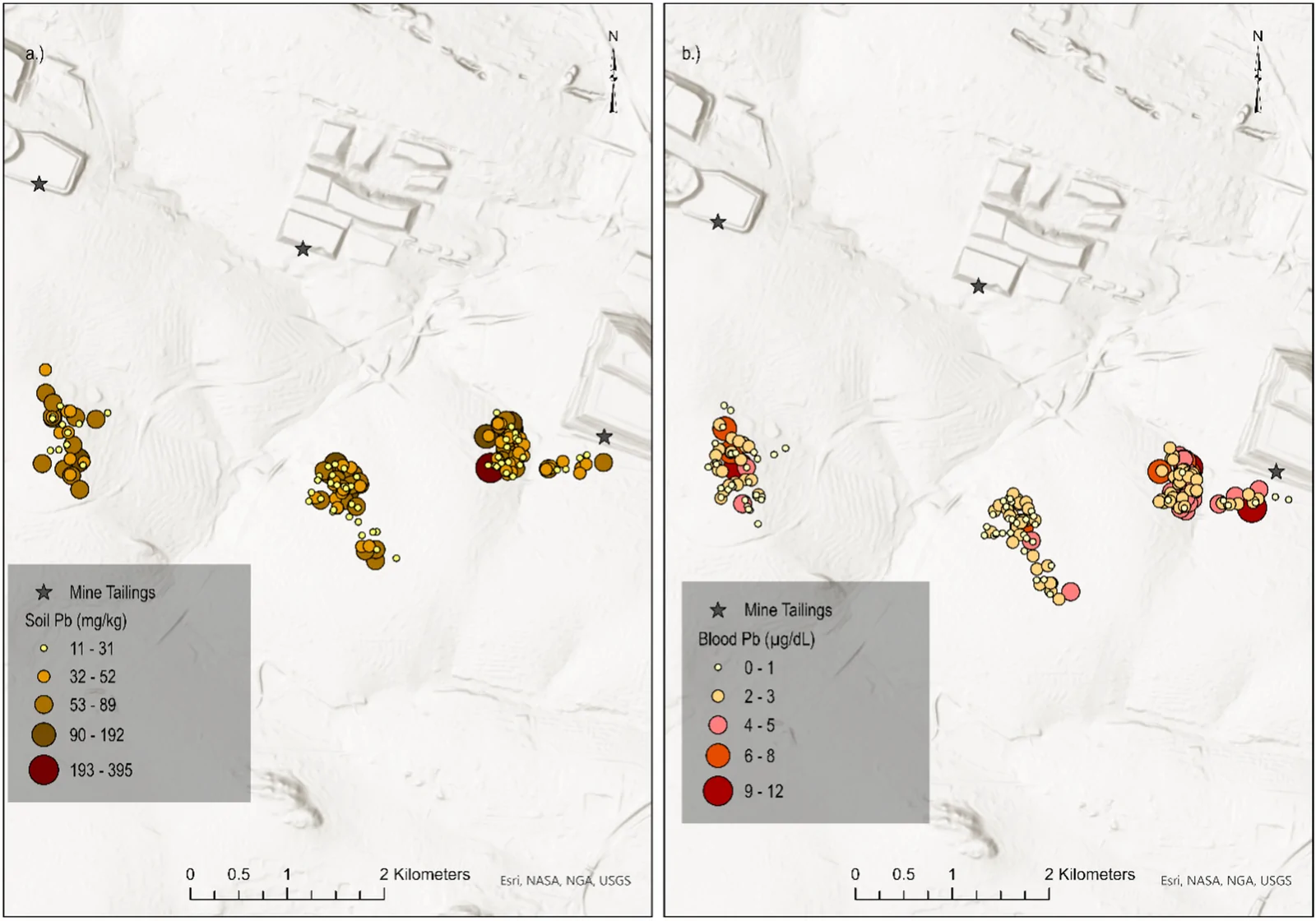
Spatial distribution of soil and blood Pb levels

Figure 2 shows statistically significant spatial clusters of elevated soil and blood Pb concentrations identified using the Getis-Ord Gi statistic. The strongest hotspots (99% confidence) for both soil and blood lead were identified in the community closest to the mine tailing (indicated by red circles), whereas communities located further away showed predominantly non-significant clustering, with only isolated cold spots (indicated by grey and blue circles). Figure 3 (Appendix) shows substantial spatial variation in soil lead concentrations, with the highest concentrations generally observed in the community closest to the mine tailing. Blood Pb levels showed a similar spatial pattern, with higher concentrations in the same community. However, some households had elevated blood Pb levels despite lower soil Pb concentrations, suggesting that soil is not the only contributor to Pb exposure. Table 2 summarises the BPAQ total score and subscales (anger, hostility, verbal aggression and physical aggression) by Pb exposure and alcohol consumption categories. The mean total BPAQ score was 79.9 ± 26.0. Mean subscale scores were 19.3 ± 5.4 for anger, 24.3 ± 6.6 for hostility, 14.9 ± 4.5 for verbal aggression, and 21.9 ± 7.8 for physical aggression. Mean verbal aggression scores were significantly higher among participants with Pb levels ≥ 5 µg/dL (17.1 ± 4.5 vs. 14.5 ± 4.5; *p* = 0.023). Alcohol use, especially binge drinking, was strongly associated with elevated total aggression scores (nptrend, *p* < 0.001). All subscales (anger, hostility, verbal aggression, physical aggression) had significantly higher scores among heavy drinkers compared to non-drinkers (all *p* < 0.001).

**Table 2:** Summary of mean total aggression score and subscales across Pb exposure and alcohol consumption categories (*N* = 199)

Outcome	All	Pb < 5 µg/dL	Pb ≥ 5 µg/dL	*p*-value^a^	No alcohol	1–4 drinks	5 + drinks^*^	*p*-value^b^
	Mean ± SD	Mean ± SD	Mean ± SD		Mean ± SD	Mean ± SD	Mean ± SD	
N	199	180 (88.4)	19 (9.5)		105 (52.8)	67 (33.7)	27 (13.6)	
Total BPAQ score	79.9 ± 26.0	78.5 ± 27.0	88.2 ± 19.1	0.139	78.3 ± 18.0	85.3 ± 17.9	101.9 ± 16.2	< 0.001
Subscales								
Anger	19.3 ± 5.4	19.4 ± 5.4	19.4 ± 5.4	0.867	18.1 ± 5.4	19.9 ± 4.9	22.8 ± 5.1	< 0.001
Hostility	24.3 ± 6.6	24.3 ± 6.7	24.3 ± 6.8	0.986	23.1 ± 6.5	24.3 ± 6.4	29.0 ± 5.2	< 0.001
Verbal aggression	14.9 ± 4.5	14.5 ± 4.5	17.1 ± 4.5	0.023	13.9 ± 4.5	14.9 ± 4.1	18.6 ± 3.8	< 0.001
Physical aggression	21.9 ± 7.8	21.6 ± 7.9	23.9 ± 6.7	0.125	19.9 ± 7.4	22.9 ± 7.9	27.0 ± 6.5	< 0.001
Blood Pb levels	2.5 ± 1.8				2.3 ± 1.9	2.5 ± 1.7	3.1 ± 1.8	0.018
Median soil Pb levels (range)	52.3 (20.5–281.0)	41.8 (20.5–281.0)	49.4 (22.9–182.2)	0.064				

^*^5 + drinks = heavy episodic drinking or binge drinking; ^a^ - Wilcoxon rank-sum test; ^b^ - Kruskal Wallis test

**Table 3:** Multivariable linear regression models of log-transformed blood Pb levels on sociodemographic, environmental, behavioural and household characteristics

Predictor	Unadjusted model	*p*-value	Adjusted model	*p*-value	
	β (SE)		β (SE)		
Sociodemographic characteristics					
Age	0.001 (0.003)	0.649			
Sex	−0.17 (0.14)	0.206			
Income					
Environmental factors					
Road proximity^*^	−0.20 (0.07)	< 0.001	−0.09 (0.05)	0.082	
Mine tailings proximity^*^	−0.39 (0.03)	< 0.001	−0.22 (0.11)	0.051	
Soil Pb concentrations^*^	0.08 (0.03)	0.007	0.08 (0.03)	0.001	
Behavioural factors					
Pica behaviour	0.35 (0.13)	0.008	0.29 (0.12)	0.017	
Tobacco smoking	0.29 (0.09)	0.002	0.20 (0.09)	0.031	
Alcohol use (Reference. No alcohol)					
1–4 drinks	0.12 (0.10)	0.259			
≥ 5 drinks (heavy episodic drinking)	0.40 (0.14)	0.006			
Household characteristics					
Major problem with dust in the home	0.14 (0.06)	0.035	0.13 (0.07)	0.043	

^*^log-transformed

Table 3 summarises the linear regression results for the predictors of log-transformed Pb levels. Higher soil Pb levels (β = 0.08, *p* = 0.001), perception that dust was a major problem in the home (β = 0.13, *p* = 0.043), tobacco smoking (β = 0.20, *p* = 0.031) and reported geophagia (β = 0.29, *p* = 0.017) were significantly positive predictors of logPb, while increased dwelling proximity to mine tailings (β=−0.22, *p* = 0.051) and roads (β=−0.09, *p* = 0.082) were marginally associated with elevated blood Pb levels.

**Table 4:** Associations of blood Pb (log-transformed) levels with total BPAQ aggression scores and subscales, including main effects and interactions with alcohol consumption categories including binge drinking

Model	Predictor	Base model β (SE)	*p*-value	Interaction model β (SE)	*p*-value
BPAQ total score	Blood lead (logPb)	3.19 (2.06)	0.123	3.60 (2.65)	0.175
	Alcohol use (reference = no alcohol)				
	1–4 drinks/day	5.79 (2.97)	0.053	4.89 (4.31)	0.259
	≥5 drinks/day	19.7 (4.14)	< 0.001	27.33 (7.89)	0.001
	Interaction term: LogPb × 1–4 drinks			1.16 (4.59)	0.800
	Interaction term: LogPb × ≥5 drinks			−7.19 (7.13)	0.268
	Age	−0.22 (0.09)	0.017	−0.23 (0.09)	0.016
Anger	LogPb	−0.02 (0.60)	0.977	0.16 (0.78)	0.841
	Alcohol use (reference = no alcohol)				
	1–4 drinks/day	2.44 (0.87)	0.006	2.65 (1.27)	0.038
	≥5 drinks/day	4.82 (1.21)	< 0.001	5.53 (2.31)	0.018
	Interaction term: LogPb × 1–4 drinks			−0.32 (1.35)	0.810
	Interaction term: LogPb × ≥5 drinks			−0.79 (2.09)	0.708
	Age	0.03 (0.03)	0.337	0.03 (0.03)	0.337
Hostility	LogPb	0.58 (0.72)	0.422	0.60 (0.93)	0.521
	Alcohol use (reference = no alcohol)				
	1–4 drinks/day	0.30 (1.04)	0.722	0.11 (1.51)	0.940
	≥5 drinks/day	4.30 (1.45)	0.003	−0.98 (2.51)	0.696
	Interaction term: LogPb × 1–4 drinks			0.26 (1.61)	0.873
	Interaction term: LogPb × ≥5 drinks			−0.98 (2.51)	0.696
	Age	−0.13 (0.03)	< 0.001	−0.13 (0.03)	< 0.001
Verbal aggression	LogPb	0.91 (0.50)	0.074	1.00 (0.65)	0.125
	Alcohol use (reference = no alcohol)				
	1–4 drinks/day	1.11 (0.72)	0.129	0.91 (1.06)	0.388
	≥5 drinks/day	4.20 (1.01)	< 0.001	5.93 (1.92)	0.002
	Interaction term: LogPb × 1–4 drinks			0.25 (1.13)	0.823
	Interaction term: LogPb × ≥5 drinks			−1.76 (1.74)	0.314
	Age	0.01 (0.02)	0.715	0.007 (0.03)	0.742
Physical aggression	LogPb	1.49 (0.86)	0.084	1.71 (1.10)	0.122
	Alcohol use (reference = no alcohol)				
	1–4 drinks/day	2.00 (1.24)	0.108	1.67 (1.80)	0.356
	≥5 drinks/day	5.16 (1.73)	0.003	8.63 (3.28)	0.009
	Interaction term: LogPb × 1–4 drinks			0.42 (1.91)	0.826
	Interaction term: LogPb × ≥5 drinks			−3.59 (2.94)	0.228
	Age	−0.11 (0.03)	0.005	−0.11 (0.04)	0.005

Linear regression models predicting aggression outcomes (Table 4) included age, alcohol use category, log-transformed blood lead (logPb), and the interaction between logPb and alcohol use. Across all aggression domains, higher alcohol consumption, particularly heavy episodic drinking i.e. ≥ 5 drinks/day category, was consistently associated with higher aggression scores (BPAQ total scores, β = 19.7, *p* < 0.001; anger, β = 4.82, *p* < 0.001; hostility, β = 4.30, *p* = 0.003; verbal aggression, β = 4.20, *p* < 0.001; physical aggression, β = 5.16, *p* = 0.003). Younger participants reported higher total aggression scores (β=−0.22, *p* = 0.022), hostility (β=−0.13, *p* < 0.001) and physical aggression (β=−0.11, *p* = 0.005). Blood Pb concentrations showed marginal positive associations with physical (*p* = 0.084) and verbal (*p* = 0.074) aggression, and was not significantly associated with total aggression, anger, or hostility. Importantly, none of the logPb × alcohol interaction terms were significant, providing no evidence that alcohol use modified the relationship between lead exposure and aggression. Overall, alcohol use was the most consistent predictor of aggression, while blood Pb contributed only modest, domain-specific associations.

## Discussion

In this study, we examined environmental and behavioural determinants of blood Pb levels and assessed whether Pb exposure was associated with aggression among adults, including potential modification by alcohol use. Compared with the original Buss and Perry (1992) college student sample, mean total aggression scores in this community-based adult population were higher (77.8 for men and 68.2 for women), suggesting elevated aggression levels in this group (Buss and Perry 1992). The present findings indicate that Pb exposure was marginally associated with aggression, with small and inconsistent effects across subscales. In contrast, alcohol consumption, particularly heavy episodic drinking, was significantly associated across all scales of aggression. This finding is consistent with previous studies linking alcohol use to heightened impulsivity and aggression (Beckwith et al. 2021; Bellinger 2008; Sontate et al. 2021). The absence of significant interaction effects suggests that alcohol use did not modify the blood Pb and aggression relationship in this study population. These results highlight that, within this community sample, aggression appears to be driven more strongly by alcohol-related behavioral risk factors than by Pb exposure.

We found that 18% of participants exceeded the CDC threshold of 3.5 µg/dL and 10% exceeded 5 µg/dL. This is a notable finding, when considered against South Africa’s current public health infrastructure, where there is no national blood lead surveillance system or routine screening program for either children or adults. Unlike the United States, where the CDC’s 3.5 µg/dL reference value results in structured follow-up, case management, and source investigation protocols, South Africa has no equivalent nationally mandated action level or clinical pathway once elevated blood lead is identified outside of formal occupational health settings. This means that the proportion of exposed individuals identified in this study would go largely undetected. This gap is particularly consequential given that these findings sit within a broader South African pattern: comparable proximity-based studies have documented similarly or more elevated blood lead levels in populations living near legacy mining infrastructure across the Witwatersrand basin (Mathee 2014; Mathee et al. 2002, 2019; Naicker et al. 2013), suggesting this is not an isolated finding but a recurring exposure pattern across Gauteng’s mining-affected communities. Historical population data from the pre-unleaded-petrol era (Mathee et al. 2006, 2019) indicate substantial national decline in background blood Pb levels over recent decades, yet the present findings suggest this decline has not eliminated meaningful residual exposure in communities close to mine tailings specifically, pointing to a source-specific rather than general population problem that broad national fuel policy alone has not resolved.

Several behavioural and environmental factors, including soil ingestion, higher soil Pb concentrations, household dust, and smoking were associated with higher blood Pb concentrations, consistent with prior research on ingestion (Lu et al. 2024; Mathee et al. 2014) and inhalation (Tan et al. 2016) pathways. In addition, increased distance from mine tailings was associated with lower blood Pb levels, suggesting a spatial gradient of exposure within the community. Gold mine tailings are a major potential source of metal contamination in nearby communities and are frequently left exposed for long periods (Romero et al. 2015; Stovern et al. 2014). Further, wind and water erosion can transport contaminated dust and soil particles well beyond the tailings’ sites, allowing the dispersion of Pb into surrounding residential areas (Romero et al. 2015). These findings highlight the role of both environmental sources and individual behaviors in Pb exposure profiles in this community.

Despite evidence of multiple exposure pathways in this study, blood Pb concentrations were not significantly associated with increased aggressive behaviour. The absence of an association may reflect the relatively low blood Pb levels observed in the study population was insufficient to detect behavioural effects. It may also indicate that the neurobehavioural effects of Pb are potentially more pronounced during developmental periods of childhood and adolescence (Naicker et al. 2012; Needleman et al. 1996; Nkomo et al. 2018; Reyes Sánchez et al. 2022; Wright et al. 2008). Although small, non-significant trends were observed for physical and verbal aggression, overall, blood Pb levels were not a major determinant of aggressive behavior. Rather, heavy episodic alcohol consumption emerged as the consistent predictor of aggression, across all subscales of aggression. Heavy episodic drinking is common among South African drinkers, with approximately 53% of adult drinkers in Tshwane reported engaging in heavy drinking (Trangenstein et al. 2018). Alcohol use is a well-established driver of aggression and interpersonal violence in South Africa (Gibbs et al. 2024; Wilson et al. 2024). Alcohol use is also a major contributor to the national burden of disease in South Africa (Matzopoulos et al. 2014, 2022; Rehm et al. 2009), and among alcohol‑attributable harms, interpersonal violence is a leading cause of disability and death among women (Abrahams et al. 2009).

Our study findings suggest that addressing harmful alcohol use as a modifiable risk factor, may be an important public health strategy in communities with ongoing lead exposure. Policies that strengthen access to screening, brief interventions, and treatment for harmful alcohol use, alongside measures to reduce environmental lead exposure, may help address multiple interacting risk factors. Environmental Pb surveillance and remediation of contaminated areas should remain a priority in communities affected by gold mine tailings. At the same time, screening and brief interventions for harmful alcohol use could address an additional modifiable risk factor identified in this study. Integrating alcohol-related interventions into primary healthcare, maternal and reproductive health services, and community-based programmes may provide practical opportunities to identify individuals at risk and provide appropriate support, particularly given the predominance of women in our study population.

Several limitations should be noted. First, the relatively small sample size limited statistical power and potentially reduced the precision of effect estimates, particularly for detecting interaction effects between blood lead levels and heavy episodic drinking. Further, convenience sampling may have introduced selection bias as participants may not fully represent all adults living near mine tailings in Gauteng province. In addition, because aggression and patterns of alcohol use often differ by sex, the overrepresentation of women may have influenced the observed associations between lead exposure, alcohol consumption, and aggression. Consequently, our findings should be interpreted with caution. Second, cross-sectional study designs cannot establish the temporal sequence between Pb exposure, alcohol use and aggression, highlighting the need for longitudinal studies that include assessment of environmental exposures to determine causal relationships. Third, alcohol consumption and aggression were self-reported, which may have introduced recall and/or social desirability bias. Heavy episodic drinking may have been underreported and aggression inaccurately reported depending on the social context. In addition, the alcohol consumption measures may not have fully captured individual drinking patterns and future studies could consider using validated screening tools, such as the Alcohol Use Disorders Identification Test (AUDIT), to improve the assessment of alcohol use. Although the BPAQ has been widely used internationally, it has not been formally culturally adapted or psychometrically validated in South African adult populations. This may limit its cultural relevance, as differences in cultural and social contexts may affect how participants interpret and respond to its items. The scale showed good internal consistency in this study population (Cronbach’s α = 0.84), however potential cultural or linguistic differences in item interpretation should be considered when interpreting the aggression findings. Residual confounding from unmeasured environmental and psychosocial factors could have influenced both Pb exposure and aggressive behavior. Communities living near gold mine tailings may experience co-exposure to other contaminants, including arsenic, cadmium, and manganese, as well as particulate matter, which were not assessed in the present study. This may have limited our ability to estimate the independent association between Pb exposure and aggressive behaviour, as potential cumulative or interactive effects of multiple environmental exposures could not be accounted for. Lastly, environmental exposure assessment was limited to residential proximity to mine tailings and soil lead concentrations, therefore, additional pathways of lead exposure may have contributed to blood lead levels. These limitations highlight the need for larger, longitudinal studies to confirm these preliminary findings.

## Conclusion

In this study, Pb exposure showed only modest and domain-specific relationships with aggression, and these were not influenced by alcohol use. Alcohol consumption, particularly heavy drinking, emerged as the most consistent predictor of aggression across outcomes. Overall, these findings suggest that, within this population, variation in aggression is driven primarily by alcohol use rather than by Pb exposure.

## Data Availability

Data available upon reasonable request.

## Appendix

See Fig. 3.
